# The *Being *of Leadership

**DOI:** 10.1186/1747-5341-6-5

**Published:** 2011-02-24

**Authors:** Wiley W Souba

**Affiliations:** 1 Vice-President for Health Affairs and Dean, School of Medicine, Dartmouth College, Dartmouth Medical School, Hanover, NH, USA

## Abstract

The ethical foundation of the medical profession, which values service above reward and holds the doctor-patient relationship as inviolable, continues to be challenged by the commercialization of health care. This article contends that a realigned leadership framework - one that distinguishes *being *a leader as the ontological basis for what leaders know, have, and do - is central to safeguarding medicine's ethical foundation. Four ontological pillars of leadership - awareness, commitment, integrity, and authenticity - are proposed as fundamental elements that anchor this foundation and the basic tenets of professionalism. Ontological leadership is shaped by and accessible through language; what health care leaders create in language "uses" them by providing a point of view (a context) within and from which they orient their conversations, decisions, and conduct such that they are ethically aligned and grounded. This contextual leadership framework exposes for us the limitations imposed by our mental maps, creating new opportunity sets for being and action (previously unavailable) that embody medicine's charter on professionalism. While this leadership methodology contrasts with the conventional results-oriented model where leading is generally equated with a successful clinical practice, a distinguished research program, or a promotion, it is not a replacement for it; indeed, results are essential for performance. Rather, being and action are interrelated and their correlated nature equips leaders with a framework for tackling health care's most complex problems in a manner that preserves medicine's venerable ethical heritage.

## Introduction

The commercialization of health care continues to undermine the deeply rooted ethical foundation of medicine as a profession that values service above reward. In this market-driven environment, write Kirch and Vernon, "physician autonomy at times appears to have become more aligned with ... the right to enhance physician revenue [such that] attention to social justice may be decreased" [1]. The four prima facie principles of medical ethics - beneficence (doing good for the patient), nonmaleficence (do no harm), respect for patient autonomy (patient choice), and justice (ensuring fair and equal treatment) - and the more recently developed Charter on Medical Professionalism [2] are routinely challenged by a health care system where the business of medicine has become business.

The issues at stake here are not esoteric or academic. Moreover, a culture in which personal gain trumps societal needs is not an illusion. The percentage of Americans who agree that higher income families should contribute more in taxes to help cover the cost of health care for lower income families decreased from 66% in 1991 to 39% in 2006 [3]. Thus, "it is important to discuss how the commercialization of medicine has fostered a distortion of emphasis among the basic tenets of medical ethics" [1]. In tackling this dilemma, physicians and other health care leaders must be front and center in the dialogue.

As central players in the social contract, physicians are granted standing, privilege, and generous remuneration with the understanding that they will adhere to the principles of service and altruism. As key players and regulators in the distribution and consumption of health care goods and services, physicians have an ethical responsibility to step back and examine their personal leadership role in creating a just society. Lee writes [4], "[H]ealth care today needs ... a new kind of leadership...; strong leaders and a [new] cultural context in which they can lead." In creating this new paradigm, sustainable success begins with transforming people first by changing their worldviews and mental maps, a process that requires leaders to spend much more time leading themselves. In the words of Peter Block, "If there is no transformation inside each of us, all the structural change in the world will have no impact on our institutions" [5].

Today, medicine's reward structure incents physicians and other leaders to focus on knowing (know-how, know-what), having (titles, power) and doing (out-performing) such that personal reward is often valued above service to others. Health care leaders can no longer ignore medicine's perverse incentives; they must confront the inefficiencies and asymmetries of delivery models, the disparity of reimbursement for services rendered, and the focus on autonomy and individualism that impede societal accountability and team behavior [1,2,6]. To restore the ethical foundation of medicine, a new model of leadership is imperative.

This article contends that a renewed context for leadership - one that distinguishes *being *a leader as the basis for what leaders know, have, and do - is central to restoring medicine's long standing ethical underpinnings. Rather than focusing on adding more degrees, titles and awards to our leadership lapel, this approach begins by "operat[ing] at the fundamental base or root level of a complete theoretical framework" [7], providing a coherent and meaningful foundation that re-contextualizes ways of being a leader and the inherent connection of that being to the actions of effective leadership. This leadership methodology is framed by several key questions: What does it mean today to "be" a leader who embodies the principles of medical ethics and professionalism? How might the way leaders and leadership "show up" in our health care organizations change if we placed as much emphasis on "being" as we do on knowing, doing, and performing? How would we redesign our approaches to leadership development?

## Leadership Meets Ontology

Leadership paradigms are grounded in several philosophical branches [8-10]. Ontology relates to the nature of being a leader, whereas axiology studies leadership values, value judgments, and ethical choices. Epistemology refers to what we can know about leading, while praxeology (sometimes referred to as methodology in the research realm) examines the leader's actions and behaviors. Theorists differ on the order for examining these assumptions but most agree with the prior nature and primacy of ontology [11-13]. Said otherwise, we are before we know we are.

Paradigms are potent because they shape how we perceive and interpret the world and act in it. In an attempt to address the plethora of healthcare's leadership challenges and offer solutions, the literature has become populated with articles that address values in medicine [14-18], the knowledge base required to be lead effectively [19-23], and the actions and results that characterize high performing leaders and organizations [24-31]. However, the *being *of leadership - what it means to *be *a leader - has not been addressed in any meaningful sort of way.

Ontology explores questions like, "When someone is being an effective leader, what is their underlying foundational 'being-ness'?" An ontological approach deals with leadership from the perspective of the way it is lived and personally experienced as opposed to any theory or concept. It does so by examining the context in which the leader's thinking, speaking, and doing arise and locate their unique character. An ontological inquiry does not replace, minimize, or undervalue leadership that is focused on results. Being and doing are distinct but inseparable; "how you categorize the world *arises together *with processes that are coordinating physical activity" [32]. Thus, the actions and interactions of leadership match the being of a leader. Action implies that it is connected to something. That something is being.

## The Philosophical Underpinnings of Being

Practically, an entity could be said to "be" only because we, as perceiving, feeling, interpreting beings experience "it" to be. Human being, however, is more than just existing. Heidegger [33] understood being as being-in-the-world, but not in the sense of a computer being (residing) on a desk or a pencil being (situated) in one's pocket. Human being is being that is aware (conscious) of, and interested in, its own being. It is being that participates in the world via a process of continuous sensemaking [34]. Through our observations, thoughts, and feelings we make meaning of our relationship with the world [35-37].

Our being and its expression emerge from our selfhood, a dynamic intersection of four dimensions: embodiment, sociality, symbolicity (linguisticality), and temporality, "essential characters which cannot be ignored if the portrait of selfhood is to maintain any coherence" [38]. As material bodies, each of us is already always in-the-world; we are social by nature, born into language, and we exist as "temporal clearings through whom world and self come into concrete existence." Our inevitable submission to existence itself has been characterized as being "thrown" into a world we do not choose, one that often seems indifferent to our trials and tribulations [33]. Faculty members at academic medical centers may experience this "thrownness" as a loss of autonomy, as an impersonal system seemingly interested solely in grant dollars and teaching hours, as a decline in income and benefits, and/or as a diminished ability to advocate for patients.

A being-centric approach to leadership does not replace a focus on actions and performance. Rather, it creates the ontological bedrock upon which the ethical foundation of medicine - acting in the best interest of the patient, safeguarding patient choice, and ensuring justice - is grounded and can be put to work. Our way of being reflects the way in which the situation we are dealing with occurs for us, which is in turn shaped by the context we bring to that situation [39]. For example, if the context we (physicians) bring to work is one that says "the uninsured are not my problem", that point of view will color the way our broken health care system occurs for us, and our actions (refusing to see non-paying patients) will be a correlate of that occurring. It is this kind of contextual "listening" that leads to an undermining of the ethics of medicine.

Contexts are constructed in language. More precisely, what is created in language provides a context that uses physicians such that they are left with new opportunity sets for being leaders who lead effectively and authentically. When we say that leadership always happens "within" a context, we take the word context to mean a set of fundamental but hidden frames of reference and assumptions that shape the way in which any situation occurs for that leader [37,39]. Context is hermeneutical - it fashions and sculpts the way we make sense of any leadership challenge or situation we are dealing with. Said otherwise, language itself, at play in the multiple interactions of human existence, adds the meanings, and, in so doing, constructs our world. An ontological inquiry maintains that our leadership worldviews - how we observe, interpret, act, and perform - are constituted in and accessible through language, rather than being given by some fixed, prelinguistic, objective reality [33,38-42].

Thus, the being of leadership is a powerful foundation that has the power to anchor the deeply-rooted ethical tenets of medicine. However, exploring this ontological realm can be foreign and disquieting. As health care providers, we can "get our arms around" our behaviors and develop or modify them, but our being is more difficult to grasp; it is less "embraceable." We don't know "being"; it's just who we are.

## The Foundational Pillars of Being a Leader

Building upon the work of Heidegger [33,41], Anton [38] and others [39,43] on selfhood, our way of being in this or that moment emerges from and is manifested in expressing and experiencing our consciousness (our awareness of ourselves and the world we live in), our commitment (to something that transcends the self), our relational congruency (authenticity with self and others), and our wholeness. A robust ontological foundation for leadership in health care should anchor its axiology, epistemology and praxis such that the ethical foundation of medicine is supported and sustained. Four ontologic pillars - awareness, commitment, integrity, and authenticity - are proposed as fundamental elements of a leadership context that has the power to use physicians and health care executives such that they are left being leaders and exercising effective leadership consistent with the basic tenets of medical ethics and professionalism. Without these four pillars serving as the bedrock upon which medicine's ethical foundation is anchored and acquires its sovereignty, all the health care reform initiatives in the world will lack any kind of meaningful integrity and sustainability.

### Awareness

The word "aware" is derived from Old English *gewær *meaning watchful, vigilant and prudent. Awareness is a state, an ability to perceive, to feel, or to be conscious of events, objects or sensations. Awareness speaks to the concept of human "being" as an observer who is perceptive and present in the moment, to include being attentive to distinguishing one's filters and blind spots, our so called ontological constraints [39,44]. In particular, it means being mindful of the limitations and distortions created by our *already-always-listening*, that ever-present voice in our head that's thinking for us and biasing us.

No human being is fully aware. While the brain processes millions of bits of sensory information every second, we are consciously aware of only a tiny fraction of them. The brain constructs small-scale models of reality that it uses to anticipate events, to reason, and to underlie explanation [45]. These mental constructs are built from deeply ingrained and internalized assumptions and generalizations that influence how we understand the world and act in it. Whenever we see anything it is filtered through a set of background assumptions and beliefs about what to look for, such that the eye sees only what the mind is primed to comprehend. Revealing the hidden and unchallenged assumptions, beliefs and frames of reference that comprise our worldview is critical to effective leadership.

An observer is any being who can be in language speaking with another (or to himself) and making distinctions [46]; a distinction is any performative linguistic act that separates (distinguishes) an entity from a background. Maturana [47] stresses the ontological primacy of observing: "Cognition has no content and does not exist outside the effective actions that constitute it. This is why nothing exists outside the distinctions of the observer.... Everything that we do becomes part of the world that we live in as we bring it forth as social entities in language." Because our filters, hidden assumptions, and other frames of reference limit and shape our possibilities for leading effectively, we must expose them. We must loosen the clasp of their handcuffs on us. We do this by making finer distinctions through language, which allows us to revise our mental maps that, prior to the moment of insight, we were stuck with. In becoming more aware, our opportunity sets for being a leader expand. In essence, we are freed up to "be".

Our mental models are not so much views and beliefs that we hold tightly as they are views and beliefs that tightly hold us. Thus, when leaders relax the hold that their engrained, taken for granted assumptions and beliefs have on them, the possibility now exists for whatever situation they are dealing with to show up for them as close as (humanly) possible to the way it actually is, such that the correlation between the occurring and their way of being and acting results naturally in their personal best for leading in that situation [39]. For example, when a physician is dealing with a homeless drug addict who has been to the emergency room seven times in the past month, *knowing *that he should be caring is very different from *being *caring in that situation. Providing care under such circumstances depends on generating a different way of being than what is likely to be his automatic way of being (impatient, callous, annoyed). The physician must experience for himself that he is not trapped with any certain way of being; rather, he can choose to be whatever way is required of him to respect the patient and honor and fulfill the doctor-patient relationship. His experience of having previously "lived through" exasperating situations creates an availability for dealing with his lack of sensitivity as a *way of being*, and then creating "being authentically sensitive and compassionate" and acting with sensitivity in the face of his aggravation.

Our awareness, derived from our five senses, is sculpted and tinted by the contexts that we carry around with us, which, in turn, shape and color the way the particular leadership challenge we must confront occurs for us. In turn, our ways of being and acting are correlated with the occurring [39]. Dijksterhuis and Bargh write:

Certainly, perception is essential for us to comprehend our environment but that does not mean that this understanding is an end in itself. Rather, understanding is a means by which we act effectively. Adaptive perception is ultimately in the service of functional behavioral responding to the environment, and comprehension and understanding are only important means to that end.... In sum, perception is for doing [48].

The power of this framework - where "learning is inherently 'situated' because every new activation is part of an ongoing perception-action coordination" [32] - is that it provides actionable access to being a leader and exercising effective leadership as one's own natural self-expression [44]. As we disassemble our deeply-engrained thoughts constructs and inherited beliefs, we can find examples in our own life where such learning shows up first-hand as a lived experience.

Heidegger compares each of us to a *clearing *(akin to what one might encounter in a forest) in which the world shows up for us. He writes, "In the midst of beings as a whole an open place occurs. There is a clearing, a lighting.... Only this clearing grants and guarantees to us humans a passage to those beings that we ourselves are not, and access to the being that we ourselves are" [41]. This clearing "is an openness, or space, in which things can be, a lit-upness in whose light things can manifest themselves as themselves.... [It] is a space of possibilities, an indefinitely complex space of possible ways for ... people to be" [49].

The encounter with a "clearing" is an encounter with *nothing*, which makes it possible for us to notice the light that we otherwise don't see [33]. This clearing refocuses the way in which we "see" who we are; rather than being defined by what we know (our expertise), have (our titles), and do (our job), we "see" who we are most fundamentally as the *being *of human being. However, this nothing of the clearing that we are is not nothing; rather, it does something; "the nothing itself noths".... [50], much as a lighting itself lights, illuminating the possibility for service and altruism.

All leaders dwell in already made clearings, some of which have become cluttered with antiquated worldviews, cognitive maps, and other hidden cultural baggage. In the act of distinguishing, we can let go of these inherited investments and outmoded beliefs. A clearing that is indeed cleared - free of ontological constraints and other limitations - creates the conditions for unlimited possibilities for being a leader. Instead of being locked into our fixed ways of being and acting, we can take a step back; the clearing that we are becomes uncluttered. There is a realization that we are not this or that person anymore; our attachments and inauthenticities fall away. This pure being-there, notes Heidegger [33], is our most basic existence. In this "standing out into nothingness," we can choose to be. "It is nothing less than a conversion from the self-centered mode of being, which always asks what use things have for us, to an attitude that asks for what purpose we ourselves exist" [51]. This "clearing out" so as to make room for being-in-the-world authentically is the ontological equivalent of cleaning out the attic.

### Commitment

The word commitment is derived from the Latin word *committere *meaning to unite, connect and bring together and the Anglo-French word *commettement*, meaning pledge, as in the obligation of oneself. At its most fundamental level, commitment is a standing for something that is bigger than oneself such that our commitment becomes who are [52]. We are rooted in others because we first learned how to be human from others. As human beings "we are articulate clearings of care over that world which exists because of us," and as "entities who care we are valuing beings and thus have concern at the very heart of our existence.... As open to being-their-possibilities, we are beings whose being remains in question.... We, as beings who exist, are both called and compelled to become who we are, and, all self-becoming is inevitably a change in our being-in-the-world" [53].

Leaders must be committed to a future that is larger than themselves [39,52]. Such a commitment is most powerful when it defines oneself and one's purpose in life while simultaneously caring for others.

[W]e cannot concern ourselves only with ourselves. Personal fulfillment cannot explicitly focus on oneself, as if selfhood were best taken care of in a literal and direct manner. Self-fulfillment comes not from a focus on one's self per se. Instead, we are concernfully absorbed by that with and which we meaningfully weave our lives...[38].

Commitments that are hollow or partial succumb and are short-lived. Real commitment means being steadfast about creating the future one stands for and being unflustered by the breakdowns that invariably show up along the way. Otherwise, the temptation to back off with the smallest derailment will be too great; after all, that envisioned future is only a possibility, not a guarantee. Commitments cannot be legislated or mandated; they have to be willingly voluntary. Leaders in health care must attend to a future that unleashes the kind of resoluteness that Gabriel Marcel characterizes as "entered upon by the whole of myself, or at least by something real in myself which could not be repudiated without repudiating the whole - and which would be addressed to the whole of being and would be made in the presence of that whole" [54]. For example, when a physician commits to providing her patients with the best care she knows how, she is taking a stand for the inviolability of the doctor-patient relationship. When a teacher commits to making available the best education possible, he is taking a stand for his students. These deepest commitments are our stand in life. Margaret Farley [55] explains:

Indeed, the history of the human race, as well as the story of any one life, might be told in terms of commitments.... At the heart of this history, however, lies a sometimes hidden narrative of promises, pledges, oaths, compacts, committed beliefs, and projected visions. At the heart of any individual's story, too, lies the tale of her or his commitments - wise or foolish, sustained or broken, fragmented or integrated into one whole.... Commitment seems, in our ordinary language, to include a notion of *willingness to do something *for or about whatever we are committed to.

As physicians, our most genuine commitments - those that are conveyed by fundamental tenets of medical ethics and professionalism - are often thwarted by our hidden assumptions and ways of doing things get in the way. Since these attachments are both diversions and obstacles, we must move them out of the way so we can make room for our stand.

### Integrity

The word integrity is derived from the Latin words *integritas *and *integer *which mean whole, entire, and intact. Integrity refers to the condition of an object, person, or organization as being whole, complete, and perfect [39]. When we talk about the integrity of a compensation model or a health care plan we mean that it is unimpaired in performing its intended functions. Integrity is a necessary condition for functionality and workability, and the resultant level of workability determines the available opportunity for performance [56]. Imagine a car whose front wheels are out of alignment; its lack of integrity results in poor function and performance, i.e., inferior workability.

For an individual, department, or organization, integrity has to do with that entity's word being whole and complete. Honoring your word means you don't break it or if you discover you cannot keep your word, you say that you will not be keeping it to those who were counting on your word and clean up any inconvenience or misunderstanding you caused by not keeping your word [39,56]. By "keeping your word" we mean doing what you said you would do - in other words, keeping your promise. Word requires transparency about what one is giving one's word to, to whom it is being given, and by when the promise given by the word will be executed. Putting integrity into practice as sticking to one's word creates the foundational opportunity for superior performance at both the individual and organizational level. In the absence of this foundation, exceptional performance is not possible and certainly not sustainable.

### Authenticity

The word "authentic" originates from the Greek word *authentes *meaning "one acting on one's own authority," i.e., that one is the accountable author of one's actions and behaviors. Being authentic is being and acting consistent with who you hold yourself out to be for others (to include who you allow others to hold you to be), and who you hold yourself to be for yourself [39,44]. Nietzsche reminds us, "The individual has always had to struggle to keep from being overwhelmed by the tribe. If you try it, you will be lonely often, and sometimes frightened. But no price is too high to pay for the privilege of owning yourself" [57].

Our desire for approval and our need to look good and measure up drives inauthentic actions and behaviors. But Hyde [58] points out that "the judgment that we are somehow in error being this way is inappropriate.... The authentic self is not one that has managed to escape the everyday thrownness of human being; rather, it is a self that has taken hold of that thrownness 'in its own way.' It has appropriated the everyday way of being, recognized it, allowed it, and owned it, rather than continuing to be owned *by *it." Thus, authenticity is about acknowledging our inauthenticity and taking responsibility for our "thrownness." Lebeau and Maclean [59] explain:

Somehow, beyond our ability to grasp, we simply found ourselves here, in this place, at this time, with these parents, with this body.... [W]e are born into something static, but are 'thrown' into something moving and dynamic; we are born 'on the run', so to speak. This 'thrownness' is not something over which we have control or which we have consciously chosen.... Thus, we embark upon our life journey from a position of *absolute *innocence and we must assume *absolute *responsibility for where we find ourselves if we are to live authentically. We could not choose how we were 'thrown' but what we can choose, and this is the crucial responsibility of being human, is *how to be *with how we were thrown.

To live authentically as a leader in health care means to be, regardless of the circumstances of our thrown 'situatedness', cause in the matter of our own lives, responsible for creating the clearing that we are. This, however, is a tall order; our attachment to the 6As - admiration, achievement, attention, authority, appearance, and affluence - is a powerful lure towards inauthenticity [36,37]. This inevitable thrownness to self-concern dislodges us from the full possibility of *being-with *others and is a major source of our inauthenticity.

Conventionality - "falling prey to the world" [60] - is perhaps the most common form of inauthenticity; we become absorbed by being-with-others or being-with-things to the extent that we no longer "live out" our authentic self. Inauthenticity can be described as being "not our own," yielding to the pressures of society, having "lost ourselves in the everydayness of existing among things and people." If you can manage to be like everyone else, you need not make your own choices. You can turn to others for advice and direction and avoid making tough decisions on your own. In other words, our authenticity (or inauthenticity) is disclosed by the way we deal with or "own" our thrownness.

For Heidegger, being human is being-in-the-world. Connected to this way of being is a way of acting such that our way of being and our way of acting are two sides of the same coin, distinguishable but inseparable. As such, our being is a "thrown projection," which has profound implications for the meaning of an authentic existence:

[W]e cannot be authentic every once and a while.... To be creatively original, to be authentic in care-taking, is to dwell habitually in the moment of decision.... Authenticity, therefore, does not exist as a single act; it exists only as a habit.... To encounter others authentically is to embody a passionate responsibility which does not simple take over the caring of others. Such a responsibility, on the contrary, operates by loosening others "from their 'they' selves" (from the inauthentic selfhood of "anyone") and thereby frees them for their own most peculiar possibilities of care. Selfhood within authentic existence fundamentally acknowledges its indebtedness to others and, in turn, is called to free others for their own possibilities [38].

It takes courage to "be-in-the-world" and deal with our "thrownness." Tillich [61] writes: "Courage as the universal and essential self-affirmation of one's being is an ontological concept. The courage to be is the ethical act in which man affirms his own being in spite of those elements of his existence which conflict with his essential self-affirmation." The courage to "be" across the full range of possibilities in life is a feature of an authentic human being. Our way of being and acting in the present is limited and shaped by the future into which we are living. Said otherwise, the future is the context for the present. Many adults are content living into their *So-So *(same 'ol, same 'ol) future, unwilling to take a powerful stand for something larger than themselves [37]. Their future becomes largely a continuation of the past. They regard being free as being free of responsibilities, rather than as an opportunity to take a stand for something larger than themselves. This disengagement is associated with submissiveness, as John Gardner [62] observed: "One of the clearest dangers in modern society is that men and women will lose the experience of participating in meaningful decisions concerning their own life and work, that they will become like cogs in the machine.... They tend to accept the spectator role and to sink into passivity."

The destabilization of medicine's ethical foundation is perpetuated by mental maps and frames of reference that de-emphasize the supremacy of the doctor-patient relationship and emphasize physician-centeredness, remuneration, and reward above service. In exposing these mental maps their hold on us is relaxed, which creates new opportunity sets for being and action (patient-centeredness, service, justice) that embody medical professionalism. The four fundamental pillars of being serve as the ontological leadership foundation upon which and from which the ethical foundation of medicine is sustained. They are factors of production, so to speak, which give leaders actionable access to being a leader and exercising effective leadership. In translating being that is aware, committed, in integrity, and authentic into action, one, a) is mindful of one's already-always-listening and other ontological constraints as an observer in life; b) takes a stand for creating a future bigger than oneself that would not otherwise happen; c) honors one's word, and; d) lives and acts consistently and genuinely with who one holds oneself to be for oneself and for others. Such a leadership framework is a powerful foundation for the ethical principles of medicine.

## Language and Being "in Action"

Our "being" as a leader is meant to be converted into action, observable and measurable as the exercise of competent leadership. Being without action is a dud; action without being is an unguided missile. For many of us, our focus as leaders is outward - on ourselves, on looking good and standing out. Fromm [63] argues that *having *and *being *are two fundamental modes of existence. The having mode "is centered on property and profit... In the having mode, one's happiness lies in one's superiority over others, in one's power.... In the being mode it lies in loving, sharing, giving."

We spend much of our lives on the run "doing" - achieving, impressing, acquiring, parleying - so we can measure up, be accepted, and be popular. We listen for confirmation that what we're saying is "right" and that we are "right." Bineham and Hyde [64] write, "Perhaps the single greatest barrier to dialogue is the pervasive human impulse to defend one's *identity - *one's *self; *and whatever one identifies with and as oneself - from any perceived threat. If one can recognize defensiveness as an inevitable aspect of human being, and can *grant it being *without feeling personally to blame for it, there is a possibility beyond it."

Leadership is a languaged experience, whereby the previously undistinguished becomes spoken and meaning-full, "released" for existence in reality. The realm of languageability includes the already spoken (both speaking and writing), the unspoken but communicated (to include the "speaking" of actions), the not yet spoken, and the unlanguagable (beyond the grasp of speaking). Whenever we observe someone being a leader or experience ourselves as leading, we "see" that person (or ourself) functioning in the sphere of language. Kegan [65] writes, "All leaders are leading language communities. Though every person, in any setting, has some opportunity to influence the nature of language, leaders have exponentially greater access and opportunity to shape, alter or ratify existing language rules.... The only question is what *kind *of language leaders we will be."

Listening is the indispensable heart of discourse in relation to others and even to oneself; "listening to...is the existential being-open of [human being] as being-with for the other [33]." The capacity to listen generously and non-judgmentally, so as to discern the context from which it is spoken, creates an access not previously available that deepens one's understanding of another person's point of view, allowing the listener to recreate for himself what is actually being said. "We do not merely speak the language - we speak by way of it. We can do so solely because we always have already listened to the language [66]."

In speaking with power, the leader's intent is to make something meaningful happen to and for the listener - an insight, a breakthrough in thinking, a sense of empowerment, or perhaps just greater clarity. We relate most powerfully with others inside of what they are committed to, inside their stand. In turn, one's listening creates what one says. Several studies indicate that female physicians are more empathic listeners, engage in greater two-way communication, and are better at encouraging patients to talk more freely than their male colleagues [67-69]. Leadership, in a very real sense, is born out of listening.

Because people's beliefs about themselves and their world inevitably are based on the interpretations they make from their own experiences and situations, each person's beliefs are different and each person can be said to live in a different reality. Moment to moment, our constructed "reality" reflects the way in which what we are dealing with (a person, a dialogue, a situation) "shows up" for us [37,70]. For example, if hospital administrators show up for us as "suits" or "bean counters," we will deal with them as such. Or, if patients occur for us as "trolls," "dirtballs," "gomers," or "widgets," our way of interacting with them and caring for them will be correlated with that occurring and contribute to a dehumanizing culture [71-75]. But the way a particular person or situation occurs for us is rarely an accurate representation of the way that person or situation actually is because our context is always already inevitably colored by biases and filters that distort what is actually there. The relationship of being to action is contextual; by "unconcealing" our biases and assumptions, we create the possibility - for a match between the way in which the challenge we are dealing with occurs for us and our way of being and acting - that results naturally in our personal best for leading in that situation.

How do we expose these veiled worldviews and mental maps so we can take a stand for "being" with greater awareness, commitment, integrity and authenticity? Guignon [76] gives us a clue:

Since language is the medium in which both self and the world can first be discovered, the meaning of 'being' must first be understood as embedded in the resources of language.... [Being] is used to capture that man, as a 'happening' or 'event,' takes a stand on his being in his every day activities.... We are always free to make something of our lives as a whole within the confines of the situations into which we are thrown.... [In] this possibility as 'being-free for its own most ability-to-be,' who we are, what we are, is determined by the concrete possibilities we take over on taking a stand on the project of our lives.

The transformative power of language resides in its ability to distinguish and shape reality. As noted by Gadamer [11], "[Language] has its real being only in the fact that the world is re-presented within it ... language has no independent life apart from the world that comes to language within it." The world we experience - the world that "shows up" for us - is largely constituted in language. In other words, language does not merely reflect reality; as a constitutive element it has the power to shape, even create, how we represent reality. It functions as a kind of lens that brings issues into sharper focus and adds clarity, allowing us to see details and "make sense" more perceptively. Language discloses a whole new world to us - a distinctively *human *world - that we would otherwise have limited access to. Rorty [77] argues that "a talent for speaking differently, rather than arguing well, is the chief instrument of cultural change."

This idea that reality is constituted in language is core to an ontological approach to leadership. Language reframes our observing, sensemaking, and feelings so we can *be *a different kind of leader. When we change our thinking and speaking, a different reality becomes available to us. Shifts in our mental maps generate new possibilities for desired actions and outcomes not previously accessible. The distinctions that we share with one another, with the intent of achieving mutual understanding, are those that occur in language. This common sensemaking is the foundation for connectivity, collaboration, and alignment, essential activities in any organization.

Thus, language is the most powerful resource we have for creating and transforming the realities we have to deal with in our lives. Transformative language doesn't describe how a situation occurs; it transforms how it occurs by rewriting the future [40]. The kinds of possibilities (personal and institutional) that leaders bring into existence are related to their ability to make distinctions as observers and listeners. Maturana [78] writes:

Distinctions take place in the domain of experiences.... It is only as different domains of languaging that physics, biology, philosophy, cooking, politics, or any cognitive domain exists.... Without observers nothing can be said, nothing can be explained, nothing can be claimed.... In fact, without observers nothing exists, because existence is specified in the operation of distinction of the observer.

Often, we cannot change the content of our lives, be it the rejection of a grant, a colleague newly diagnosed with cancer, or a child with a learning disability. But while we can't do much about what we know, we can alter the way we know it. We have the freedom to alter the way we distinguish the situations that occur in our lives; we can shift the context. Content is always observed within a linguistic context, one created by distinctions. Only by means of language can you and I lead ourselves, each and every day, to become the wiser, more enlightened, and more evolved human beings that we are intended to become. Anton writes, "Speech is as much for individuals as for others. This means that thought is no less social than speech is thoughtful. Why? Because persons make sense to themselves as they make sense to others: by speech. I speak to share thought with myself.... Language does not simply 'represent' thought; speaking or listening - to myself or to others - accomplishes thought [79]." We transform ourselves into better leaders through language.

## Being and Transformation

Intimately linked to an ontological context for leadership in health care is the ethical foundation that naturally emerges. Being and ethicality in medicine are correlated. When one is being a leader in medicine who takes a stand for the inviolability of the doctor-patient relationship, one naturally places service above reward. We cannot compare delivering health care to delivering the mail. Illness and suffering are not just biologic problems to be solved by research and technology - they are also enigmas which can point out our limitations, vulnerabilities, and frailties, and our link with one another. Ultimately, both our understanding of and approach to the physician-patient relationship depends on our ontology that frames what it means to be human, of the purpose and meaning of human life. To fracture that relationship by exchanging compassion and justice in favor of the marketplace is something none of us would want for ourselves. McLaughlin writes, "The rush to transform patients into units on an assembly line demeans medicine as a caring as well as curative field, demeans the respect due every patient and ultimately demeans illness itself as a significant human condition" [80].

The four ontological pillars - awareness, commitment, integrity, and authenticity - constitute a framework for leadership that provides physicians and health care executives with a context within which and from which their conversations, decisions, and conduct are naturally connected to the basic tenets of medical ethics and professionalism. While one could make a case for other framework elements (e.g. compassion, honesty, fairness), the four foundational pillars discussed in this paper encompass them. In his magnum opus, *A Theory of Justice*, Harvard political philosopher John Rawls [81] poses a provocative question: What kind of world would you want to be born into if you did not know what your social status would be, what your intelligence and natural abilities would be, or what your opportunities in life would be? Indeed, this is the kind of world we are each born into. Mindful, discerning people, argues Rawls, would answer this critically important question by replying that they would opt for a world that was compassionate and just. The ontological framework discussed in this paper is consistent with Rawls' writings.

The transformation of health care begins with a shift in our understanding of what it means to be a leader. When a physician creates for himself a future for healthcare to which he is committed that is bigger than his own agenda, that future "uses" him in that it provides a context from which his correlated actions are altruistic, service-oriented, and attentive to social justice. When a hospital executive distinguishes his already-always-listening that says "we want to avoid uninsured patients", it creates the possibility for taking a stand for patient autonomy and justice. When a health care provider gives his word to the Charter on Professionalism [2], he makes a promise (that uses him) to maintain patient confidentiality, improve quality of and access to care, and to practice medicine so as to ensure the just distribution of finite resources.

Many of the circumstances that physicians and other health care leaders have been "thrown" into - the corporatization of medicine, decreasing reimbursement for services rendered, increasing time constraints, and more regulations - are seriously taxing the underlying code of medical ethics and professionalism [1,3,43,82-87]. As observers, we can ask: What distinctions (thoughts, mindsets, feelings, experiences, and assumptions) are disclosed to me in my thrownness? How does this situation that I've been thrown into occur for me? Am I willing to observe it differently? Guignon [76] writes: "The outcome of our lives is something that matters to us; it is *at stake *or *in question *for us. And because the point of our lives is an issue for us, we always take some concrete stand on our Being-a-whole." In taking a stand for our being, we can ask: From a position of awareness, commitment, integrity, and authenticity, what is the optimal orientation for my "being-in-the-world" in this situation, right now?

Good leaders help us "see" our filters and blind spots by creating insights that make revisions of our mental maps possible. This opens the door to meaningful personal change. The moment we are willing to challenge a deeply held assumption or a habitual way of behaving, the possibility of transformation becomes real. Ontologically, this leadership approach entails an inward journey of self-discovery and reinvention. In the cognitive, affective and behavioral neuroscience realm, it is about letting go of our obsolete mental maps that hold us back from exercising more effective leadership. Electrophysiologically, this new learning involves re-wiring of neural circuits. Functionally, this leadership journey enhances our workability, performance, and joy in life.

What if the future of your medical center or college of medicine lay in your hands? And what if all that you could do (or not do) as a leader depended on your way of being? What distinctions would you want to emerge from your being as an observer? What ways of being would preserve the ethical foundation of medicine? What kind of language leader would you want to be? Because leadership exists in the sphere of language and the future exists only in language, leadership is called for when a desired future (one that fulfills the legitimate and rightful concerns of the relevant parties) can only be achieved by creating a new linguistic context, one that creates openings and possibilities (previously unavailable) that give people being and action in the present that results in the realization of that desired future [39].

Who we are being as a leader in medicine is our stand in life. Our stand is an expression of our deepest commitments. It is connected to action that focuses on improving the lives of others - our patients, our students, our peers, and our community. How we are being as a leader is aware, committed, in integrity, and authentic. What we are being as a leader is joy. Tillich [61] writes, "Joy is the emotional expression of the courageous Yes to one's own true being." Where we are being is in our thrownness, wherever life takes us, in spite of the uncertainty. Why we are being a leader is because we are called to be. The call to "be", notes Heidegger [33], "is precisely something which *we ourselves *have neither planned nor prepared for nor voluntarily performed, nor have we ever done so. 'It' calls, against our expectations and even against our will. On the other hand, the call undoubtedly does not come from someone else who is with me in the world. The call comes *from *me and yet *from beyond me and over me*."

## About the author

Wiley "Chip" Souba (pronounced Show-buh) serves as Vice-President for Health Affairs and Dean of the Medical School at Dartmouth College. He holds a faculty appointment as Professor in the Dept of Surgery and in the Tuck School of Business. Dr. Souba is an AOA graduate of the University of Texas Medical School in Houston, where he also did his surgical residency. He earned a Doctorate in Science in Nutritional Biochemistry at the Harvard School of Public Health and subsequently did a fellowship in Surgical Oncology at the MD Anderson Hospital and Tumor Institute in Houston. Dr. Souba earned his MBA at the Boston University College of Business. He is recognized for his innovative approaches to developing leaders and leadership. He writes and lectures regularly on topics such as health care leadership challenges, personal and organizational transformation, leading oneself, barriers to effective leadership, and the language of leadership.

Author details: Office of the VP and Dean, Medical Administration, 1 Rope Ferry Road, Hanover, NH 03755

## Competing interests

The author declares that they have no competing interests.
